# Rapid identification of an Arabidopsis NLR gene as a candidate conferring susceptibility to *Sclerotinia sclerotiorum* using time‐resolved automated phenotyping

**DOI:** 10.1111/tpj.14747

**Published:** 2020-04-21

**Authors:** Adelin Barbacci, Olivier Navaud, Malick Mbengue, Marielle Barascud, Laurence Godiard, Mehdi Khafif, Aline Lacaze, Sylvain Raffaele

**Affiliations:** ^1^ Laboratoire des Interactions Plantes Micro-organismes (LIPM) Université de Toulouse INRAE CNRS 24 chemin de Borde Rouge - Auzeville CS 52627 F31326 Castanet Tolosan Cedex France

**Keywords:** quantitative disease resistance, fungal pathogen, *Sclerotinia sclerotiorum*, NBS‐LRR, plant phenotyping, technical advance

## Abstract

The broad host range necrotrophic fungus *Sclerotinia sclerotiorum* is a devastating pathogen of many oil and vegetable crops. Plant genes conferring complete resistance against *S. sclerotiorum* have not been reported. Instead, plant populations challenged by *S. sclerotiorum* exhibit a continuum of partial resistance designated as quantitative disease resistance (QDR). Because of their complex interplay and their small phenotypic effect, the functional characterization of QDR genes remains limited. How broad host range necrotrophic fungi manipulate plant programmed cell death is for instance largely unknown. Here, we designed a time‐resolved automated disease phenotyping pipeline enabling high‐throughput disease lesion measurement with high resolution, low footprint at low cost. We could accurately recover contrasted disease responses in several pathosystems using this system. We used our phenotyping pipeline to assess the kinetics of disease symptoms caused by seven *S. sclerotiorum* isolates on six *A. thaliana* natural accessions with unprecedented resolution. Large effect polymorphisms common to the most resistant *A. thaliana* accessions identified highly divergent alleles of the nucleotide‐binding site leucine‐rich repeat gene *LAZ5* in the resistant accessions Rubezhnoe and Lip‐0. We show that impaired *LAZ5* expression in *laz5.1* mutant lines and in *A. thaliana* Rub natural accession correlate with enhanced QDR to *S. sclerotiorum*. These findings illustrate the value of time‐resolved image‐based phenotyping for unravelling the genetic bases of complex traits such as QDR. Our results suggest that *S. sclerotiorum* manipulates plant sphingolipid pathways guarded by LAZ5 to trigger programmed cell death and cause disease.

## Significance Statement

We developed an image‐based method to measure plant quantitative disease resistance over time with high resolution and throughput. We applied it to reveal Arabidopsis* LAZ5* as a susceptibility gene to the necrotrophic fungal pathogen *Sclerotinia sclerotiorum* using only six plant genotypes. *LAZ5* belongs to a family of typical resistance genes, suggesting that *S. sclerotiorum* exploits classical resistance pathways to cause disease.

## INTRODUCTION

The fungal pathogen *Sclerotinia sclerotiorum* is the causal agent of *Sclerotinia* stem rot (SSR), also designated as white mold disease, on numerous crop and vegetable species, including rapeseed, soybean, sunflower, and tomato. *S. sclerotiorum* can be among the most damaging pathogens of rapeseed and soybean when conditions are favourable (Peltier *et al.*, 2012; Derbyshire and Denton‐Giles, 2016). *S. sclerotiorum* penetrates plant tissues through wounds, natural openings, or actively forming compound appressoria (Bolton *et al.*, 2006). It employs a typical necrotrophic strategy to colonize host tissues, rapidly triggering plant cell death (Kabbage *et al.*, 2015; Mbengue *et al.*, 2016). As genetic sources of resistance to SSR are lacking for most crop species, adapted cultural practices, the use of fungicides, and biological control methods are frequently employed to limit damages due to *S. sclerotiorum* (Derbyshire and Denton‐Giles, 2016). Instead of a clear demarcation between resistant and susceptible genotypes, plants challenged with *S. sclerotiorum* generally show a continuum of resistance levels designated as quantitative disease resistance (QDR) phenotype (Perchepied *et al.*, 2010; Roux *et al.*, 2014). The molecular bases of QDR in plants remain largely elusive (Poland *et al.*, 2009; Roux *et al.*, 2014). Whereas resistance (R) genes mediating complete disease resistance all belong to the nucleotide‐binding site leucine‐rich repeat (NLR) family, genes underlying QDR discovered to date span a broad range of molecular functions (Poland *et al.*, 2011; Roux *et al.*, 2014; Corwin and Kliebenstein, 2017). Molecular function of genes associated with QDR include for instance transporters (Krattinger *et al.*, 2009), kinases (Fu *et al.*, 2009; Huard‐Chauveau *et al.*, 2013), peptidases (Poland *et al.*, 2011; Badet *et al.*, 2017b), or actin‐related proteins (Moscou *et al.*, 2011). NLRs (Debieu *et al.*, 2015; Lee *et al.*, 2016) and signalling components typically associated with R‐mediated resistance (Iakovidis *et al.*, 2016) can also mediate QDR, illustrating the tight integration of QDR and R‐mediated immunity components.

Because of their complex interplay and their small phenotypic effect, the functional characterization of QDR genes is challenging. With large collections of mutant lines available, studies in the model plant *A. thaliana* enable the rapid functional characterization of individual candidate QDR genes (Huard‐Chauveau *et al.*, 2013; Corwin *et al.*, 2016; Rajarammohan *et al.*, 2018). Fully quantitative readouts are often required to reveal the contribution of individual *A. thaliana* genes to QDR against *S. sclerotiorum*, such as ethylene and ROS detection (Perchepied *et al.*, 2010; Zhang *et al.*, 2013), lesion area measurements, and estimation of fungal biomass (Zhang *et al.*, 2013; Badet *et al.*, 2017b). To this end, quantitative image analysis can be used as a proxy to evaluate disease severity (Baranowski *et al.*, 2015; Mutka *et al.*, 2016; Karisto *et al.*, 2017; Badet *et al.*, 2017b) and the impact of stress on plant fitness (Chen *et al.*, 2014; Nelson *et al.*, 2018; Czedik‐Eysenberg *et al.*, 2018). Increasing the accuracy and robustness of such quantitative phenotyping often involves increasing the number of measurements. This allowed for instance to reveal the role of individual effectors from the bacterial pathogen *Xanthomonas axonopodis* pv. *manihotis* in virulence (Mutka *et al.*, 2016). High‐throughput image‐based plant phenotyping proved valuable in crop breeding and for research purposes (Araus and Cairns, 2014; Fahlgren *et al.*, 2015; Coppens *et al.*, 2017). There is therefore a need to improve our ability to generate large datasets of quantitative plant disease measurements at low cost, low footprint, and reduced human intervention (Czedik‐Eysenberg *et al.*, 2018). Because it is generally non‐destructive, image‐based disease measurement also gives access to the dynamics of disease progression. This enabled for instance to study the spatial and temporal distribution of pathogens in plants tissues (Mutka *et al.*, 2016) and distinguish between infected and non‐infected plants long before qualitative symptoms are visible (Czedik‐Eysenberg *et al.*, 2018).

In spite of remarkable progress in methods and technology in recent years, fundamental advances in plant pathology enabled by automated plant phenotyping are still relatively limited. A method for the image‐based phenotyping of plant disease symptoms combining high throughput, high resolution, low footprint, and low cost is currently lacking. This design is crucial to identify novel QDR genes and advance our conceptual understanding of this complex trait. Here, we present the Navautron system, an integrated hardware and software solution capable of automatically calculating disease lesion area over time on detached leaves inoculated by pathogens causing necrotic lesions. As a proof of concept, we compared the susceptibility of sunflower genotypes inoculated by the fungal pathogen *S. sclerotiorum* and *Nicotiana benthamiana* leaves inoculated by the oomycete pathogen *Phytophthora infestans*. In its current design, the Navautron system is able to capture 800 *A. thaliana* leaves per m^2^. Its automatic leaf and lesion tracking features allow discriminating disease symptom dynamics with differences in lesion doubling time, as small as 12 min, with nearly no human intervention.

To illustrate how the Navautron system can advance our fundamental understanding in plant pathology, we used it to demonstrate that *A. thaliana* NLR gene *LAZ5* (At5g44870), but not its close relative *constitutive shade avoidance 1* (*CSA1*), confers susceptibility to *S. sclerotiorum*. For this, we precisely assessed the kinetics of disease caused by seven *S. sclerotiorum* strains on six *A. thaliana* natural accessions. We found that the speed of lesion growth was highly dependent on the host plant genotype and a good indicator of QDR level. We hypothesized that large effect polymorphisms common to the two most resistant *A. thaliana* accessions, but absent from the four other accessions, would point towards disease susceptibility genes. We identified highly divergent alleles of *LAZ5* in the resistant accessions Rubezhnoe and Lip‐0, while identical alleles existed in the other *A. thaliana* accessions analyzed. As expected, two *LAZ5*‐deficient mutant lines in the Col‐0 genetic background showed enhanced QDR to *S. sclerotiorum*, whereas plants mutated in the closely related *CSA1* gene responded like the wild‐type. Considering that the ectopic activation of *LAZ5* triggers cell death (Palma *et al.*, 2010), our results suggest that the broad host range necrotrophic fungus *S. sclerotiorum* exploits this R gene induced plant cell death to its benefit.

## RESULTS

### Quantification of disease resistance by automated time‐resolved image analysis

**Figure 1:**
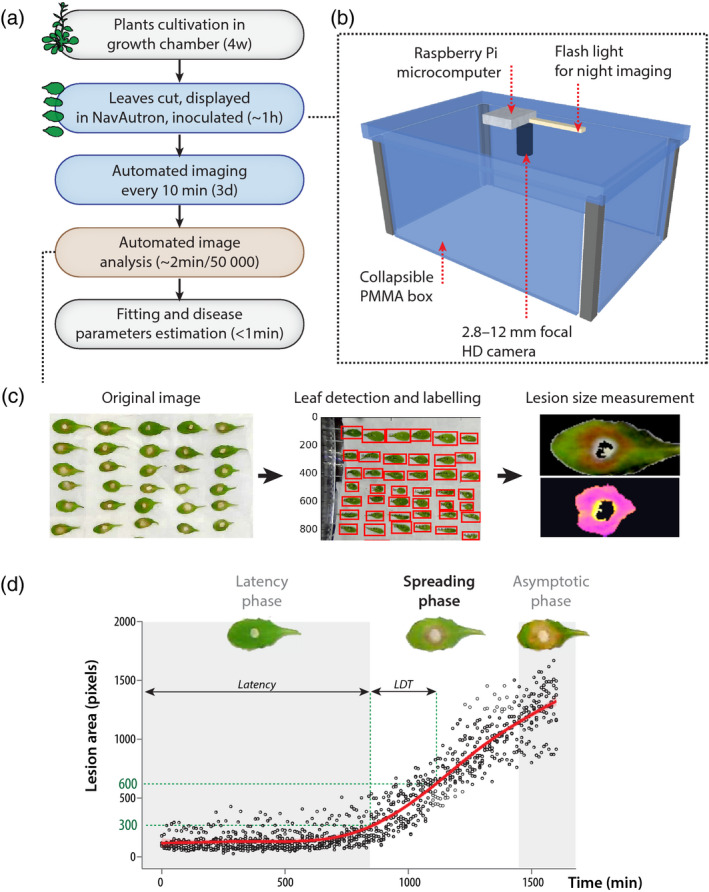
Analysis of quantitative disease resistance (QDR) against *Sclerotinia sclerotiorum* with the Navautron system. (a) Pipeline describing the experiments reported in this manuscript. Detached leaves were analyzed with the Navautron through automated imaging, automated image analysis, curve fitting, and QDR parameters estimation. Approximate duration for each step is indicated (d, days; w, weeks). (b) The Navautron setup: Each Navautron consists of a transparent plastic box (poly(methyl‐methacrylate), PMMA) equipped with a Raspberry Pi microcomputer, a high‐definition (HD) camera and a LED flash light. (c) The three major steps of the automated image analysis. (d) Typical kinetics of *S. sclerotiorum* disease lesion development on *A. thaliana*, illustrating the latency phase, spreading phase, and asymptotic phase. Characteristic values are the duration of latency phase and the lesion doubling time (LDT). Data shown correspond to values collected on five leaves of *A. thaliana* Col‐0, the red curve shows fitted average.

To generate massive, quantitative, and kinetic measurements of disease caused by *S. sclerotiorum*, we designed mobile imaging cabinets (Navigable automatized phytotron, Navautron) and the associated automatized image analysis pipeline for detached leaves (Figure 1a). A typical phenotyping experiment on detached leaves inoculated by *S. sclerotiorum* involves: (i) plants cultivation in growth chambers, (ii) leaves cutting, inoculation and display, (iii) automated imaging every 10 min over 3 days (36 h are usually sufficient), (iv) automated recognition of disease lesions on individual leaves, and (v) estimation of disease parameters. A Navautron unit (Figures 1b and S1) is composed of a poly(methyl‐methacrylate) box of internal dimensions 53 (length) × 38.6 (width) × 45 (height) cm equipped with a Raspberry Pi microcomputer, a high‐definition (HD) camera, and a light‐emitting diode (LED) flashlight. The low footprint of this system allows using it in various environments such as growth chambers, growth cabinets, and incubators. It can be assembled from widely available standard parts making it very affordable (typically under 300€ per unit). A single Navautron unit allows imaging simultaneously up to 120–270 detached leaves of 4‐ to 5‐week‐old *A. thaliana* (Figure S1). Alternatively, although not optimized for this application, the system can image 24–32 whole *A. thaliana* plants simultaneously. A high‐definition webcam located at the centre of the lid of Navautron boxes is set to image the bottom tray carrying inoculated leaves every 10 min. We designed a bioinformatics pipeline to extract automatically the size of disease lesions and parameters describing disease kinetics. The first step of the analysis pipeline consists in a python script called INFEST (kINematic oF lESion development) which calculates lesion areas for each leaf in a series of images (Figure 1c and Boxes 1 and 2). The script requires: (i) a time series of images taken by a Navautron box, all located in the same folder, and (ii) a layout text file providing an identifier and bounding rectangle coordinates for each leaf. For a typical experiment with *A. thaliana* and *S. sclerotiorum* (120 leaves imaged for 36 h), a single Navautron will yield *c.* 26 000 lesion measurements with almost no human intervention.

Box 1 **Installation guide for INFEST, the Navautron image analysis tools**

A complete tutorial and updates can be found at https://github.com/A02l01/INFEST. Infest requires python and conda installed on your machine. Major steps of the installation procedure are:

1. Clone the infest repository

$ git clone https://github.com/A02l01/INFEST.git

- Create and activate the INFEST conda environment using the yaml file

$ conda env create ‐n INFEST ‐f env_Infest.yml

- Analyze pictures contained in a directory after activating INFEST conda environment

$ conda activate INFEST

$ python infest.py path_to_picture.

Numbers of the first and last picture to analyze could be specified e.g.

$ python infest.py path_to_picture ‐f 0 ‐l 400.

**Infest command line**

**usage: infest.py [‐h] [‐f FIRST] [‐l LAST] mpath**



**positional arguments:**


mpath Path to the directory containing pictures


**optional arguments:**


‐h, ‐‐help show this help message and exit

‐f FIRST, ‐‐first FIRST Number of the first picture

‐l LAST, ‐‐last LAST Number of the last picture

Box 2 **User guide for disease lesion analysis with the INFEST script**

Pre‐requisites: Image files composing a time series must all be located in the same directory with images named by an integer e.g. 1.jpg to N.jpg in chronological order (https://github.com/A02l01/tuto/tree/master/data_tuto/pictures for example). Gaps between pictures are admitted.

1. Determine bounding rectangles coordinates for each leaf (in pixels). To this end, we recommend opening one image file in ImageJ software, tracing a rectangular ‘Region of Interest’ around each leaf and retrieving the corresponding coordinates. For accurate lesion size measurement, leaves position must remain identical through the whole duration of the experiment (usually the case when images are acquired through the Navautron system) and that possible distortion due to camera lens are corrected.
2. Create a grid_layout/ subdirectory in the image directory
3. Create a grid_layout.layout file within the grid_layout/ directory. This should be text file containing one line per leaf to be analyzed, and on each line five items separated by tabs: (i) a leaf identifier, (ii) the minimum coordinate of the bounding rectangle on y‐axis, (iii) the minimum coordinate of the bounding rectangle on x‐axis, (iv) the maximum coordinate of the bounding rectangle on y‐axis, and (v) the maximum coordinate of the bounding rectangle on x‐axis. For an example of the layout file, see the Github page of the INFEST script here: https://github.com/A02l01/tuto/tree/master/data_tuto/pictures/grid_layout. **Note that placing leaves at the same position when setting up experiments greatly accelerates this step.**
4. Run the INFEST script in conda environment with command

$ conda activate INFEST

$ python infest.py path_to_picture ‐f first_picture ‐l last_picture

where

path_containing_images is the full path of the directory containing pictures (e.g. /home/foo/bar/). Pictures must be named by an integer corresponding to time (e.g. 1.jpg, 100.jpg). path_containing_images is a positional argument.

‐f first (optional, default min(image_name)) is an integer corresponding to the first image to consider.

‐l last (optional, default max(image_name)) is an integer corresponding to the last image to consider.

- The output will be an analysis of the .txt file created in the path_containing_images directory containing three columns: the Id of leaf, the time extracted from pictures name and the lesions size.
- Deactivate conda environment

$ conda deactivate

A complete tutorial and updates can be found at https://github.com/A02l01/INFEST

We distinguished three major phases along the kinetics of lesions development (Figure 1d): (i) a latency phase, during which no necrotic lesion was detected, (ii) a spreading phase, during which the area of necrotic lesions grew exponentially to reach (iii) an asymptotic phase, when lesions have spread over the whole leaf surface. The overall kinetics are therefore depicted by a logistic function. The asymptotic phase was dependent only on leaf size which sets the value of the asymptote and then did not provide information on disease progression. Although exponential, the spreading phase can be approximated classically by a polynomial function through Taylor’s development. The disease lesion doubling time (LDT, in minutes) is deduced from the slope of the lesion size curve during the spreading phase. Importantly, the LDT is determined at the beginning of the spreading phase, before lesions reach the edge of infected leaves, to reduce bias due to leaf shape and size. The LDT is the characteristic value for the infection since it is sufficient to characterize completely the spreading phase.

The computation of an accurate LDT required the post‐treatment of the kinematics of lesion obtains by image analysis (Box 3). To limit the potential effect of noise and the rate of image acquisition on the computation of the LDT, kinematics of lesion development was first fitted by a 4‐degree polynomial. Note that the fit of the lesion kinematics allows also the computation of the LDT even if a few data points are missing. As *A. thaliana* leaves had areas over 1000 pixels, the LDT was computed as the time required by the infection to increase from 300 to 600 px. For the purpose of comparing kinetics, we recommend the transformation by natural logarithm Log(LDT), yielding a distribution closer to normality and increasing the validity of the associated statistical analyses.

Box 3 **User guide for the computation of the LDT by the fit_INFEST script**

usage: fit_INFEST.py [‐h] [‐ft FIRST] [‐g] path_in path_out.

*positional arguments*

path_inthe path to the file containing temporal data computed by INFEST

path_outthe path to the file containing LDT and Latency

*optional arguments*

‐h, ‐‐help show this help message and exit

‐ft FIRST, ‐‐first FIRST the first time to consider to compute the LDT

‐g, ‐‐graph monitoring the fit of the curve

Pre‐requisites:

an analyse.txt file computed by the infest script.

1. activate INFEST conda environment

$ conda activate INFEST

- Run fit_INFEST script

$ python fit_INFEST.py path_to_picture/analyse.txt path_to_somewhere/ldt.txt ‐g ‐ft 400

### Assessment of the Navautron system versatility

**Figure 2:**
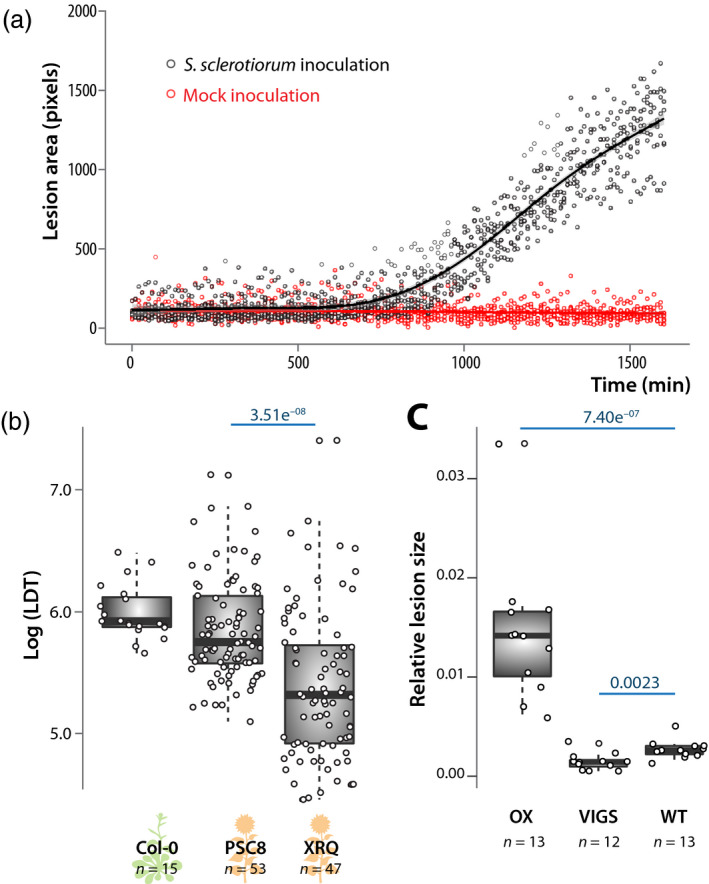
Assessment of sensitivity and versatility of the Navautron system for quantifying plant disease symptoms. (a) Kinetics of disease lesion development of leaves inoculated by *S. sclerotiorum* (black) and mock inoculated (sterile agar plug, red). Data shown correspond to values collected on five leaves of *A. thaliana* Col‐0, lines show fitted average. (b) Lesion doubling time (LDT) measured on leaves of *A. thaliana* Col‐0 accession and sunflower PSC8 and XRQ genotypes after inoculation by *Sclerotinia sclerotiorum*. LDT was measured on *n* = 15 to 53 mature leaves per genotype in three independent biological replicates. Statistical difference between LDT on the two sunflower genotypes was assessed by Student’s *t*‐test with *P*‐value indicated in blue. (c) Relative size of necrotic lesions measured on leaves of *Nicotiana benthamiana* plants overexpressing the REM1.3 remorin protein (OX), silenced for rem1.3 by virus‐induced gene silencing (VIGS), and wild‐type (WT), after inoculation by *Phytophthora infestans*. Measurements were performed on 12 or 13 infection sites per genotype. Statistical difference between LDT was assessed using a Wilcoxon’s test with *P*‐value indicated in blue. Boxplots show 1st and 3rd quartiles (box), median (thick line) and the most dispersed values within 1.5 times the interquartile range (whiskers).

Our lesion detection algorithm is based on the intensity of the red channel relative to the green channel in leaf images, which proved successful in detecting disease lesions caused by *S. sclerotiorum* on *A. thaliana*. To verify that this approach is not sensitive to the physiology of detached leaves, we compared lesion detection in *A. thaliana* leaves inoculated by *S. sclerotiorum* and by a sterile agar plug as a mock control (Figure 2a). The signal collected from mock inoculated leaves remained very low for the whole duration of the experiment, allowing to discriminate unambiguously disease symptoms from general stress in detached leaves. To verify that our lesion detection algorithm is not sensitive to the type of leaf we first analyzed the kinetics of *S. sclerotiorum* lesion development on two sunflower (*Helianthus annuus*) genotypes. Previous field trials have reported that *S. sclerotiorum* induced larger lesions on sunflower genotype XRQ than on genotype PSC8, which was selected for capitulum resistance to *S. sclerotiorum* (Bert *et al.*, 2002). We quantified *S. sclerotiorum* LDT on *A. thaliana* Col‐0 accession and sunflower genotypes XRQ and PSC8 using the Navautron system (Figure 2b). Lesions were detected reliably on both plant species with the same settings. We obtained an average Log(LDT) = 6.00 (doubling time 6.91 h) on *A. thaliana* Col‐0. Log(LDT) was 5.84 (doubling time 6.31 h) on the resistant sunflower genotype PSC8 and 5.40 (doubling time 4.54 h) on the susceptible sunflower genotype XRQ (Wilcoxon’s test *P*‐value = 3.51e^−08^). Second, we analyzed images of *Nicotiana benthamiana* leaves inoculated by the oomycete pathogen *Phytophthora infestans* obtained from (Bozkurt *et al.*, 2014), taken with a conventional digital camera. We applied the INFEST algorithm to measure the size of lesions 8 days post inoculation on lines accumulating the REM1.3 protein to different levels (Figure 2c). Lesions were on average *c.* 5.2 times larger on lines overexpressing REM1.3 (OX) than on wild‐type (WT) (Wilcoxon’s test *P*‐value = 7.40e^−07^). By contrast, lesions were *c.* 1.8‐fold smaller on plants silenced for REM1.3 (VIGS) than on wild‐type (*P*‐val = 0.0023). These results correctly recapitulated the findings by that REM1.3 accumulation enhances *P. infestans* infection. We conclude that our plant phenotyping setup is suitable to track necrotic lesions caused by diverse pathogens over time on multiple plant species and should prove useful to progress in our understanding of the mechanisms underlying plant–pathogen interactions.

### Grouping of plant and fungal genotypes according to QDR phenotypes

**Figure 3:**
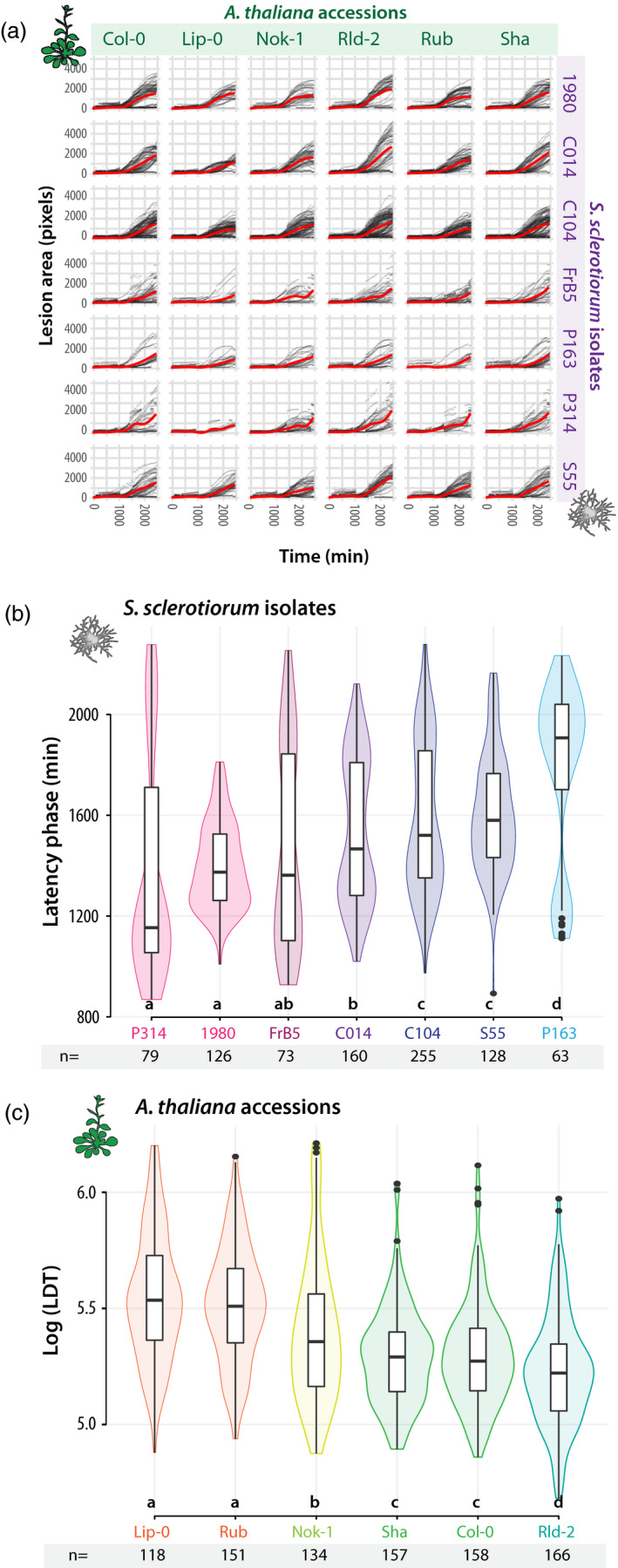
Characteristic values describing disease symptom dynamics in the interaction between seven *Sclerotinia sclerotiorum* isolates and six *A. thaliana* accessions. (a) Kinetics of disease lesion development for 42 different combinations of *A. thaliana* natural accessions (columns) and *S. sclerotiorum* isolates (lines). Red curves show smooth fitting curves for 1500 to 12 250 measurements. (b) The duration of latency phase (Y‐axis) was mostly dependent on *S. sclerotiorum* isolates (x‐axis), ranked from the most (P314) to the least virulent (P163). Duration of the latency phase was measured *n* = 63 to 255 times for each isolate. (c) The lesion doubling time (LDT, y‐axis) was mostly dependent on *A. thaliana* accessions (y‐axis), ranked from the most (Lip‐0) to the least resistant (Rld‐2). LDT was measured *n* = 118 to 158 times on each accession. Letters and colours indicate groups of significance determined by post hoc pairwise *t*‐tests. Boxplots show 1st and 3rd quartiles (box), median (thick line) and the most dispersed values within 1.5 times the interquartile range (whiskers).

To document the diversity of QDR phenotypes in the *A. thaliana*–*S. sclerotiorum* pathosystem, we quantified the area of necrotic lesions caused by seven isolates of *S. sclerotiorum* (Badet *et al.*, 2017a) on detached leaves from six natural accessions of *A. thaliana* (Figure 3a). In the 42 interactions tested, the duration of the latency phase and the speed of lesion size increase during the spreading phase were independent (*R* ^2^ = 0.07). The duration of the latency phase was mainly dependent on *S. sclerotiorum* genotype (*P* = 1.2 × 10^−21^) with a significant but weaker effect of plant genotype (*P* = 2.95 × 10^−4^). This suggested that the latency phase was primarily determined by fungal strains virulence. Necrotic disease lesions were detected after a latency phase of 1 to 1.5 days in average, mostly determined by *S. sclerotiorum* genotypes. Post hoc pairwise *t*‐tests with Benjamini–Hochberg *P*‐value correction (Benjamini and Hochberg, 1995) revealed four groups of *S. sclerotiorum* isolates with distinct latency phases (Figure 3b). Isolates 1980 and p314 had the shorter latency phase (average 22.6 and 23.2 h), isolates FrB5 and C014 had an intermediate latency phase (average 24.4 and 25.5 h), isolates C104 and S55 had a longer latency phase (average 26.4 and 26.7 h) and isolate P163 had the longest latency phase (29.8 h). The smallest significant difference in latency phase that could be detected in this experiment was about 1h. The ranking of isolates according to their latency phase was identical on all *A. thaliana* accession and therefore not dependent on the plant genotype.

By contrast with latency, Log(LDT) was mostly dependent on the *A. thaliana* genotype (*P* = 7.16.10^−37^) and weakly function of *S. sclerotiorum* isolates (*P* = 2.39.10^−3^). We conclude that the Log(LDT) provides an appropriate measure of QDR to *S. sclerotiorum*. The average LDT ranged between 3.08 and 4.24 h, corresponding to Log(LDT) of 5.22 to 5.54 (Figure 3c), mostly determined by *A. thaliana* genotypes. Post hoc pairwise *t*‐tests with Benjamini–Hochberg *P*‐value correction (Benjamini and Hochberg, 1995) revealed three groups of resistance (Figure 3c). With an average Log(LDT) 5.55 and 5.51, Lip‐0 and Rubezhnoe (Rub) accessions were substantially more resistant than Nok‐1, Col‐0 and Shahdara (Sha) (average Log(LDT) 5.40, 5.30 and 5.30 respectively), whereas Rld‐2 exhibited the highest susceptibility to any *S. sclerotiorum* strain tested (average Log(LDT) 5.23). The smallest significant difference in LDT that could possibly be detected in this experiment was 13.3 min (Log = 0.069). The ranking of *A. thaliana* accessions according to LDT was identical with all *S. sclerotiorum* genotypes. Although the precise ranking of accessions based on LDT differs from the ranking obtained previously with disease severity index on whole plants with *S. sclerotiorum* strains S55 (Perchepied *et al.*, 2010) and 1980 (Badet *et al.*, 2017b), we consistently found Lip‐0 and Rub among the most resistant accessions and Rld‐2 among the most susceptible ones.

### Identification of candidate QDR genes in *A. thaliana* by an association approach

**Figure 4:**
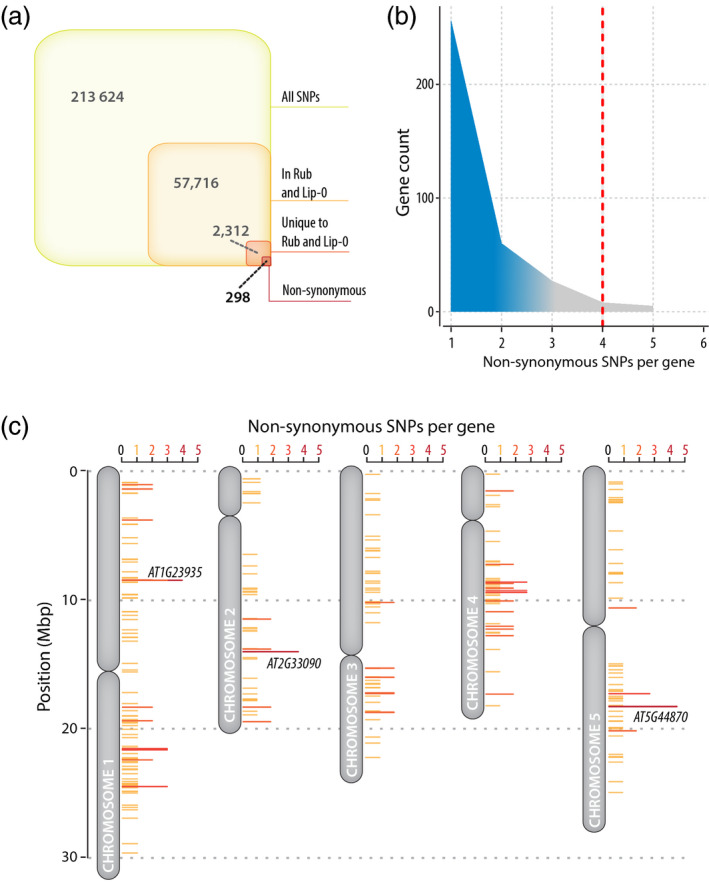
Distribution of single nucleotide polymorphisms (SNPs) in selected *A. thaliana* accessions and identification of candidate disease‐relevant genes. (a) Distribution of SNPs genotyped by (Atwell *et al.*, 2010) through our pipeline for finding candidate disease‐relevant genes. There were 2312 SNPs common to Lip‐0 and Rub but not present in Nok‐1, Rld‐2, and Sha, among which 298 were non‐synonymous SNPs. (b) Number of non‐synonymous SNP per gene in the list of 298 SNPs identified in (a). We report on the three genes including at least four non‐synonymous SNPs in Lip‐0 and Rub but no SNP in Nok‐1, Rld‐2, and Sha (red dotted line). (c) Map of *A. thaliana* chromosomes showing genes with non‐synonymous SNPs in Lip‐0 and Rub but no SNP in Nok‐1, Rld‐2, and Sha. Genes discussed in the text are labelled on the figure.

We hypothesized that enhanced QDR in Lip‐0 and Rub accessions could result from disruptive mutations in QDR‐relevant genes. To rapidly pinpoint such candidate QDR genes, we screened the genome of *A. thaliana* for genes harbouring non‐synonymous mutations both in Lip‐0 and Rub accessions but not in any of the other four accessions analyzed (Nok‐1, Col‐0, Sha, and Rld‐2) (Figure 4a). For this, we screened 213 624 positions genotyped in the six *A. thaliana* accessions selected (Atwell *et al.*, 2010). We found a total 57 716 single nucleotide polymorphisms (SNPs) present both in Lip‐0 and Rub, among which 2312 were not found in Nok‐1, Rld‐2, or Sha (unique to Rub and Lip‐0). Among those, 298 were non‐synonymous SNPs unique to Lip‐0 and Rub. The density of non‐synonymous SNPs unique to Lip‐0 and Rub accessions per gene followed an exponentially decreasing distribution (Figure 4b). Three genes harboured at least four non‐synonymous SNPs in Lip‐0 and Rub but not any non‐synonymous SNP in Nok‐1, Sha, or Rld‐2 (Figure 4c and Table 1). This included *AT1G23935*, encoding an uncharacterized protein with similarity to apoptosis inhibitory proteins, *AT2G33090*, encoding an uncharacterized member of the transcription elongation factor IIS family, and *AT5G44870* encoding *LAZ5*, a disease resistance protein of the TIR‐NBS‐LRR (NLR) family with similarity to *RPS4* and *CSA1* (Palma *et al.*, 2010). In a suppressor screen, single point mutations in *LAZ5* resulted in a dominant negative phenotype (Palma *et al.*, 2010). *LAZ5* is expressed and induced 2.32 fold during the infection of *A. thaliana* by *S. sclerotiorum* (Badet *et al.*, 2017b), and was given the highest priority for functional validation.

**Table 1:** List of genes harbouring at last four non‐synonymous SNPs in Lip‐0 and Rub but none in Nok‐1, Sha, and Rld2

Gene ID	Description	Chr.	SNP pos.	From	to	SNP type	Gene LFC
AT1G23935	Apoptosis inhibitory protein	1	8462159	C	G	not_syn	3.93 *P* = 0
		1	8462342	C	G	not_syn	
		1	8463263	T	C	not_syn	
		1	8465002	G	A	not_syn	
AT2G33090	Transcription elongation factor (TFIIS) family protein	2	14035515	C	G	not_syn	2.97 *P* = NA
		2	14035545	G	T	not_syn	
		2	14035721	C	G	not_syn	
		2	14035944	C	G	not_syn	
AT5G44870	Disease resistance protein (TIR‐NBS‐LRR class) family (LAZ5)	5	18115125	T	C	not_syn	1.21 *P* = 0
		5	18115243	A	G	not_syn	
		5	18116419	C	G	not_syn	
		5	18116946	A	G	not_syn	
		5	18118097	T	A	not_syn	

Chr., chromosome; LFC, Log_2_ fold gene of gene expression upon inoculation by *S. sclerotiorum*, with the adjusted *P*‐value for differential expression. NA, not applicable; pos. position.

### Disruption of *LAZ5* increases quantitative resistance to *S. sclerotiorum*

**Figure 5:**
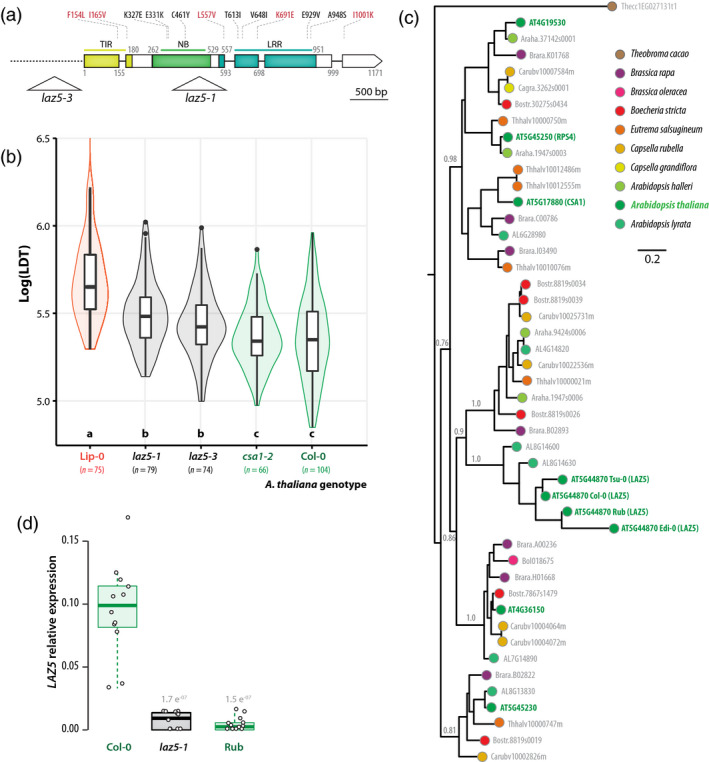
Disruption of the NLR gene *LAZ5* reduces lesion doubling time upon *Sclerotinia sclerotiorum* challenge. (a) Schematic map of the *LAZ5* gene showing the position of T‐DNA insertion in the *laz5‐1* and *laz5‐3* mutant lines (triangles). Exons are shown as boxes, introns as plain lines, upstream non‐coding region as a dotted line. Domains encoded by exons are colour coded and labelled TIR, NB, and LRR. Positions are given as amino acid numbers. Non‐synonymous mutations known in Lip‐0 allele are indicated above boxes, in red when present in (Atwell *et al.*, 2010), in black otherwise. (b) Lesion doubling time (LDT, Y‐axis) in the most resistant accession Lip‐0, two *laz5* mutant lines, the *csa1‐2* mutant, and Col‐0 wild‐type. LDT was measured *n* = 74 to 104 times on each accession. Letters and colours indicate groups of significance determined by post hoc pairwise *t*‐tests. (c) Phylogenetic relationship of LAZ5 and its 42 closest homologues in the phytozome 12.1 database. LAZ5 closest homologue outside of the *Brassicaceae* family (*Theobroma cacao* 1EG027131) was included as outgroup, and alleles from *A. thaliana* Edi‐0, Rub, and Tsu‐0 natural accessions to represent infraspecific diversity. The tree obtained by a maximum likelihood analysis is shown, with the number of substitution per site used as branch length, and branch support determined by an approximate likelihood‐ratio test (grey labels). Terminal nodes are colour coded according to plant species. (d) Relative expression of the *LAZ5* gene determined by quantitative RT‐PCR in healthy plants. Values shown correspond to three independent biological samples analyzed through four technical repeats each. Statistical difference from expression in Col‐0 plants was assessed with Student’s *t*‐tests. Boxplots show 1st and 3rd quartiles (box), median (thick line) and the most dispersed values within 1.5 times the interquartile range (whiskers).

To test for a role of *LAZ5* as a susceptibility gene against *S. sclerotiorum*, we analyzed the phenotype of two *LAZ5* insertion mutant lines in the Col‐0 background during infection with *S. sclerotiorum* 1980. The *laz5‐1* null mutant (SALK_087262C) carries a T‐DNA insertion in the second exon of *LAZ5* and shows dominant suppression of autoimmune cell death in an *acd11‐2* background (Palma *et al.*, 2010). The *laz5‐3* mutant (SALK_068316) carries a T‐DNA insertion *c.* 300 bp upstream of *LAZ5* start codon (Figure 5a andData S1). Using our Navautron system, we also determined the resistance to *S. sclerotiorum* in *csa1‐2* mutant plants defective in *AT5G17880*, a TIR‐NBS‐LRR gene closely related to *LAZ5* (Faigón‐Soverna *et al.*, 2006). As opposed to *laz5‐1*, the *csa1‐2* mutant showed enhanced susceptibility to avirulent strains of the bacterial pathogen *Pseudomonas syringae* (Faigón‐Soverna *et al.*, 2006; Palma *et al.*, 2010). Consistent with our previous measurements (Figure 3), the average Log(LDT) was 5.35 on Col‐0, similar to the Log(LDT) on the *csa1‐2* mutant (Student’s *t*‐test *P*‐value = 0.68) (Figure 5b). The *laz5‐1* and *laz5‐3* mutants had an average Log(LDT) of 5.5 and 5.44, significantly higher than Col‐0 (*P*‐value = 1.3e^−05^ and 0.01 respectively). This represented an average LDT of 3.51 h in Col‐0 and 4.08 h in *laz5‐1*, equivalent to a 16% gain of QDR in the *laz5‐1* mutants compared with wild‐type. The automated analysis of 74–104 plants per genotype thanks to the Navautron setup allowed assessing this quantitative variation robustly. In agreement with our previous measurements (Figure 3), the Lip‐0 accession showed an average Log_10_(LDT) of 5.7, significantly higher than the *laz5* mutants (*P*‐value = 3.0e^−07^ and 1.6e^−10^).

In the dataset from (Atwell *et al.*, 2010), five non‐synonymous SNPs were found in the TIR domain, the LRR domain and the C‐terminal extension of *LAZ5* (Figure 5a). Data from http://signal.salk.edu/atg1001/ revealed seven additional non‐synonymous SNPs in the NB and LRR domains of the Lip‐0 allele of *LAZ5* and indicated that several accessions, including Rub and Edi‐0, harboured large deletions within *LAZ5* coding sequence. To document natural diversity at the *LAZ5* locus, we analyzed phylogenetic relationships between *A. thaliana* Col‐0 *LAZ5* and its 42 closest homologues retrieved from the Phytozome 12.1 database (Goodstein *et al.*, 2012) (Figure 5c and Data S2). We included the closest homologue retrieved outside the Brassicaceae family (*Theobroma cacao* 1EG027131) as outgroup, as well as alleles from *A. thaliana* Edi‐0, Rub, and Tsu‐0 natural accessions to represent infraspecific diversity. *LAZ5* belonged to a monophyletic clade clearly separated from its closest *A. thaliana* relatives *CSA1*, *RPS4*, *At4G19530*, *At4G36150,* and *At5G45230*. LAZ5 clade was only represented in *A. thaliana* and *A. lyrata*, while its sister clade is represented in most *Brassicaceae* species but not *A. thaliana*. Together with long tree branches in *LAZ5* clade, this indicates strong sequence divergence among Arabidopsis accessions at the *LAZ5* locus. We used quantitative RT‐PCR to assess the expression of three *LAZ5* alleles in healthy *A. thaliana* plants (Figure 5d and Data S1). We found that LAZ5 expression was strongly impaired in the *laz5.1* mutant (*c.* 13‐fold reduction, Student’s *t*‐test *P*‐value 1.7e^−07^) and in Rub natural accession (*c.* 21.3‐fold reduction, *P*‐value 1.5e^−07^). These results indicate that reduced expression of mutant and natural alleles of *LAZ5* correlate with enhanced QDR to *S. sclerotiorum* and suggest that the Rub allele of *LAZ5* diverged functionally from its Col‐0 counterpart. We conclude that disruption of the *LAZ5* gene contributes to the enhanced QDR against *S. sclerotiorum* measured in Lip‐0 and Rub accessions compared with Col‐0. This identifies *LAZ5* as a TIR‐NBS‐LRR gene candidate conferring susceptibility to *S. sclerotiorum* through its Col‐0 allele.

## DISCUSSION

Quantitative disease resistance is a complex trait governed by multiple genes of small to moderate effect (Poland *et al.*, 2009; Roux *et al.*, 2014; Corwin and Kliebenstein, 2017). Revealing the phenotypic contribution of such small‐effect genes challenges our ability to quantify precisely and robustly the level of QDR in diverse plant genotypes. In *Botrytis cinerea* interaction with *A. thaliana*, most plant genes were associated with QDR against a specific *B. cinerea* isolate (Corwin *et al.*, 2016), emphasizing pathogen genetic diversity as a determinant of QDR phenotype. Previous studies used disease index (Perchepied *et al.*, 2010; Rajarammohan *et al.*, 2018), ethylene production (Zhang *et al.*, 2013), camalexin production (Corwin *et al.*, 2016), and lesion area (Corwin *et al.*, 2016; Badet *et al.*, 2017b) to quantify QDR against necrotrophic fungi in natural accessions and mutants of *A. thaliana*. Here we chose lesion area measurement to assess QDR for being: (i) a continuous parameter (by contrast with discrete disease indices), (ii) non‐destructive and therefore giving access to disease kinetics, and (iii) amenable to automated image‐based measurement, opening the way to user‐independent high‐throughput quantification.

For the experiments reported in this manuscript, we conducted *S. sclerotiorum* inoculations and QDR measurement on *A. thaliana* detached leaves to reach 120 samples analyzed with a single Navautron cabinet. This approach does not provide a complete characterization of plant QDR as discrepancies may exist between detached leaves and whole plant disease resistance (Liu *et al.*, 2007), lesion area may differ from area colonized by the fungus (Kabbage *et al.*, 2015). This could explain differences in the ranking of *A. thaliana* accessions according to resistance between this study and (Perchepied *et al.*, 2010). Our Navautron pipeline gives access to the kinetics of symptoms development during plant‐fungal pathogen interactions, which offers several advantages over single end‐point measurements. First, it allows uncoupling LDT and latency duration. Since latency appeared mostly independent from plant genotype in our analysis, LDT provides a more direct measurement of plant QDR potential than end‐point measurements. This approach would allow untangling plant and pathogen genetic factors contributing to quantitative immunity (Corwin *et al.*, 2016). Second, the resolution and data richness offered by time‐resolved image‐based phenotyping allows detecting small phenotypic effects that are not accessible to classical approaches, revealing for instance the virulence function of single pathogen effectors (Mutka *et al.*, 2016). Here we could detect robustly variations as small as 1h (about 3.9%) in latency period and 12 min (about 5.24%) in LDT. Finally, LDT is independent of leaf shape and size and allows comparison of QDR in plants with contrasted leaf architectures.

In this initial study, *A. thaliana* accessions ranked identically for their LDT against seven *S. sclerotiorum* isolates, arguing against specific plant–pathogen genotype interactions below the species level. Previous studies reported genetic determinants of plant QDR specific of pathogen genotypes (i.e. evidence for ‘pathotypes’) in the interaction of *B. cinerea* with *A. thaliana* interaction (Corwin *et al.*, 2016) and *S. sclerotiorum* with *Brassica napus* and *B. juncea* (Ge *et al.*, 2012; Barbetti *et al.*, 2014). These pathotypes may result from the combined effect of fungal genetic determinants of latency duration and plant determinants of LDT. Future experiments will expand the diversity of *S. sclerotiorum* isolates and *A. thaliana* accessions screened by time‐resolved phenotyping to search for specific plant × pathogen interactions affecting LDT.

Like most pathogens, fungi secrete molecules, often termed ‘effectors’, to manipulate host physiology and cause disease (Doehlemann *et al.*, 2014; Lo Presti *et al.*, 2015; Schornack *et al.*, 2009). Some pathogen effectors are recognized by specific plant R genes, leading to a rapid and efficient immune response designated as effector‐triggered immunity (ETI) (Dodds and Rathjen, 2010). In some plant–pathogen interactions, plant resistance is triggered only in plant genotypes carrying an R gene enabling the specific recognition of an avirulence effector produced by the pathogen, according to a gene‐for‐gene model (Flor, 1956). However, this model rarely applies to plant interactions with necrotrophic pathogens. ETI often involves the rapid programmed death of host cells at the site of infection, a process designated as the hypersensitive response (HR) (Mur *et al.*, 2008). Although very efficient to control the spread of biotrophic pathogens, the HR can instead favour the colonization of plants by necrotrophic fungi (Govrin and Levine, 2000). A number of necrotrophic fungal pathogen specialized to infect a few plant species evolved effectors designated as host‐specific toxins (HSTs) that trigger cell death specifically in some plant genotypes (Friesen *et al.*, 2008; Oliver and Solomon, 2010). Typical HST examples are the victorin peptides produced by the necrotrophic Victoria Blight fungal pathogen *Cochliobolus victoriae*. Victorin is critical for *C. victoriae* virulence, which is mediated through the specific recognition by the plant LOV1 protein belonging to the NLR class (Lorang *et al.*, 2007). Functional and structural analyses of LOV1 suggest that it corresponds to a typical R gene that has been hijacked by *C. victoriae* to trigger cell death and facilitate infection (Lorang *et al.*, 2012; Wolpert and Lorang, 2016). The wheat *Tsn1* gene belongs to the NLR class and recognizes the ToxA peptide produced by the necrotrophic fungus *Stagonospora nodorum*, conferring effector‐triggered susceptibility (ETS) to this pathogen (Faris *et al.*, 2010). Another effector produced by *S. nodorum*, SnTox1, triggers programmed cell death when recognized by the wheat gene *Snn1* encoding a wall‐associated kinase (WAK) (Liu *et al.*, 2012; Shi *et al.*, 2016). WAKs contribute to resistance against biotrophic and hemibiotrophic fungal pathogens (Zuo *et al.*, 2015; Hurni *et al.*, 2015) providing another example of a biotrophic pathogen defence mechanism hijacked by a specialized necrotrophic fungus. Our knowledge on whether and how broad host range necrotrophic fungi manipulate plant programmed cell death remains nevertheless limited.

The analysis of HR‐deficient *Arabidopsis thaliana* mutants suggested that the broad host range necrotrophic fungi *S. sclerotiorum* and *Botrytis cinerea* can benefit from HR cell death (Govrin and Levine, 2000). Similarly, inactivation of the BIK1 kinase enhances *A. thaliana* resistance to avirulent bacterial pathogens but increases susceptibility to *B. cinerea* and *Alternaria brassicicola* (Veronese *et al.*, 2006). Several molecules secreted by broad host range necrotrophic fungi trigger cell death in plants. Examples include *B. cinerea* endo‐arabinase BcAra1 (Nafisi *et al.*, 2014), xyloglucanase BcXYG1 (Zhu *et al.*, 2017), the *S. sclerotiorum* necrosis, and ethylene‐inducing peptide SsNEP1 (Dallal Bashi *et al.*, 2010) or *Alternaria tenuissima* Hrip1 protein elicitor (Kulye *et al.*, 2012). Cell death induction by these elicitors is often due to direct toxic effects on plant cells independently of the manipulation of plant programmed cell death (Lenarčič *et al.*, 2017). Conversely, the *A. thaliana* aspartyl protease APCB1 cleaves the BAG6 cochaperone, triggering autophagy, and restricting *B. cinerea* colonization (Li *et al.*, 2016). This findings points towards a role for autophagy cell death in resistance to necrotrophic fungi (Lai *et al.*, 2011; Lenz *et al.*, 2011) and highlight the complex role of plant programmed cell death in the interaction with necrotrophic pathogens.

Time‐resolved image‐based phenotyping with the Navautron system allowed identifying *LAZ5* as a susceptibility gene to *S. sclerotiorum*. *LAZ5* belongs to the TIR‐NBS‐LRR family and is required for *ACD11*‐mediated cell death (Palma *et al.*, 2010). Overexpression of *LAZ5* results in hypersensitive cell death, while *LAZ5* mutant alleles suppress the autoimmune phenotype of *acd11* mutants (Palma *et al.*, 2010). This analysis identifies *LAZ5* as a prime example of a TIR‐NBS‐LRR gene probably controlling susceptibility to a broad host range necrotrophic fungus. Whether effector molecules secreted by *S. sclerotiorum* interfere directly or indirectly with *LAZ5* function remains to be determined. Testing for enhanced resistance of *laz5* mutants to diverse *S. sclerotiorum* isolates and necrotrophic fungal species should be a promising future direction to address this question.

Broad host range fungal pathogens invest a substantial fraction of their cellular energy in secreted proteins to counter plant defences and extract nutrients from plant cells (Badet *et al.*, 2017a). Manipulation of the *LAZ5* pathway may be part of *S. sclerotiorum* strategy to trigger plant cell death actively. Autoimmune mutants such as *acd11* were proposed to be altered in functions guarded by NB‐LRR genes such as *LAZ5* (Palma *et al.*, 2010; Tong *et al.*, 2017). *ACD11* encodes a ceramide‐1‐phosphate transfer protein, which led to the hypothesis that LAZ5 could guard sphingolipid metabolic pathways targeted by pathogen effectors (Simanshu *et al.*, 2014). Necrosis and ethylene‐inducing peptide 1–like (NLP) are toxins secreted by diverse plant pathogens that target plant sphingolipids (Lenarčič *et al.*, 2017). *S. sclerotiorum* produces NLPs (Dallal Bashi *et al.*, 2010) the activity of which could be guarded by *LAZ5*. *A. thaliana* mutants in the dihydrosphingosine‐1‐phosphate lyase *AtDLP1* are more resistant to *B. cinerea* (Magnin‐Robert *et al.*, 2015) supporting the view that sphingolipids are important mediators of QDR against necrotrophic fungi. Alternatively, *LAZ5* activity could be costly for plants so that the inactivation of this gene would be beneficial even in the absence of pathogen. However, we did not detect growth defects in *laz5* mutants, arguing against this hypothesis. The similar LDT measured in *csa1‐2* and wild‐type plants upon *S. sclerotiorum* inoculation also pleads for a relatively specific role of *LAZ5* in QDR against this fungus. *CSA1* and *RPS4* are two TIR‐NBS‐LRR genes closely related to *LAZ5* that confer resistance to avirulent strains of the bacterial pathogen *Pseudomonas syringae* (Gassmann *et al.*, 1999; Faigón‐Soverna *et al.*, 2006), whereas *LAZ5* does not (Palma *et al.*, 2010), supporting *LAZ5* specific function.

Inactivation of *LAZ5* contributed *c.* 30% of the reduced LDT in Lip‐0 accession, indicating that Lip‐0 harbours other genetic variants positively affecting QDR. Lip‐0 also shows high QDR against *B. cinerea*, *Plectosphaerella cucumerina,* and *Fusarium oxysporum* (Llorente *et al.*, 2005), making it a useful natural resource for studying the determinants of QDR. The Navautron system is flexible allowing for variations and improvements. Future experiments will include imaging whole plants to detect putative spatial bottlenecks to disease progression, age‐related factors associated with QDR and trade‐offs between QDR and plant growth.

## EXPERIMENTAL PROCEDURES

### Plant material and cultivation

Six natural accessions of *A. thaliana* were chosen to cover the range of resistance to *S. sclerotiorum* (Perchepied *et al.*, 2010): Lip‐0 (CS76542; ecotype ID: 8325), Rubezhnoe‐1 (CS76594; 7323), Nok‐1 (CS78282; 7270), Shahdara (CS78397; 6962), Col‐0 (CS76778; 6909), and Rld2 (CS78349; 7457). Plants were grown at 22°C, 9 h light period at 120 μmol m^−2^ sec^−1^ during 4 weeks before inoculation. Arabidopsis insertion mutant lines in the Col‐0 background were obtained from the Nottingham Arabidopsis Stock Centre (NASC). Mutant lines in *LAZ5* (*AT5G44870*) were SALK_087262C (*laz5‐1*) and SALK_068316 (*laz5‐3*), the *csa1‐2* mutant line was SALK_023219C. Homozygous T‐DNA insertion was verified by PCR for each line following recommendations from the NASC website. Sunflower genotypes PSC8 and XRQ were obtained from the Centre of Biological Resources for sunflower (crb.tournesol-toulouse@inrae.fr) and grown in 22°C, 9 h light for 16 days before inoculation.

### Fungal cultivation and inoculation

The seven isolates of *S. sclerotiorum* used in this work are described in (Badet *et al.*, 2017a). Four isolates (C014, C104, P314, and P163) were obtained from a rapeseed field population collected in Blois (France) in 2010, isolate FrB5 was obtained from (Vleugels *et al.*, 2013), isolate S55 was obtained from (Perchepied *et al.*, 2010) and isolate 1980 is *S. sclerotiorum* reference strain (Derbyshire *et al.*, 2017). *S. sclerotiorum* isolates were grown on Potato Dextrose Agar (PDA) plates for 5 days at 23°C in the dark before inoculation. In total, 288–308 combinations of *A. thaliana* accessions and *S. sclerotiorum* isolates were distributed in two Navautrons in a fully randomized design. Leaves were cut with a scalpel blade at the time of inoculation, placed adaxial face up on wet paper towel overlaid at the bottom of Navautrons. Inoculations were performed as described in Badet *et al. *(2017b), using PDA plugs of 5 mm diameter (*A. thaliana*) and 8 mm diameter (sunflower) colonized by the fungus placed upside down on the adaxial surface of leaves (mycelium in contact with the leaf). The first true leaves were used for experiments with sunflower. The experiment was repeated three times independently and results from the three replicates were combined for analysis. Statistical analyses were performed using R software and the ‘car’ library for type II anova and pairwise *t*‐test with Benjamini–Hochberg *P*‐value correction (Benjamini and Hochberg, 1995; R Core Team, 2014). Distributions of LDT were normalized by natural logarithm transformation. Plots were made using the ‘ggplot2’ library (Wickham, 2010).

### Design of the Navautron phenotyping cabinets

Five‐mm‐wide PMMA plates were cut on a Trotec Speedy 500 Laser cutting machine according to plans provided as Data S1. Holes were drilled at the centre of the upper panel to place full HD 1080p USB cameras with 2.8–12 mm focal length (model ELP‐USBFHD05MT‐FV‐F1 manufactured by ELP, China). The cameras were plugged to Raspberry Pi 3 Model B motherboards equipped with Waveshare 3.2 inch thin‐film transistor touchscreens. Autofocus and automatic white balance were disabled. For each experiment, pictures were taken every 10 min during 4 days and stored on secure digital cards. To obtain uniform light exposure during image acquisition, a LED flash light is turned on for 5 sec during both day and night time. Day and night images could then be processed identically. Navautrons were placed in Percival AR‐41L2 growth chambers at 22°C with *c.* 90% humidity under 9 h light period.

### Image analysis pipeline

The area of necrotic disease lesions was computed by the INFEST python script available at https://github.com/A02l01/INFEST. Briefly, the script relies on a thresholding method based on colour analysis using the scikit‐image python image analysis toolbox (van der Walt *et al.*, 2014). The method splits images of an infected leaf into three layers corresponding to the background, the lesion, and the leaf. The ‘background’ corresponded to pixels with a saturation value superior or equal to 0.3 in HSV colour space. The lesion corresponded to pixels with red component higher than green value in RGB colour space. The area of disease lesions was computed as the sum of pixels in the ‘lesion’ layer. The remaining pixels were attributed to the ‘leaf’ layer. Image analysis allowed the collection of lesion area values over time. Every kinetics was fitted by a 4‐degree polynomial regression to limit the effect of noise on the computation of the LDT. Fit of the kinematics of lesions development was computed by the numpy polytfit library. The latency time and LDT were extracted directly from the fit. The latency time was defined as the time needed to reach a lesion area of 300 pixels. The LDT was computed as the difference between the time when lesion area reached 600 pixels and the time when lesion area reached 300 pixels. Note that these values have been set for the present data analysis but can be changed easily in python script provided at https://github.com/A02l01/INFEST. Kinetics that exhibited excessive signal to noise ratio were automatically excluded from the analysis as follows: we excluded leaves of size <300 pixels and leaves with a maximum lesion size less than the variance of lesion size during the latency period. About three leaves were excluded per Navautron representing <2.5% of all leaves. Images of *N. benthamiana* leaves 8 days post inoculation by *P. infestans* were obtained from (Bozkurt *et al.*, 2014). Detached leaves were inoculated by droplets of *c.* 500 zoospores of *P. infestans* strain 88069 tdtomato. Overexpressing plants were stably transformed with a p35S‐YFP:StREM1.3 construct (Raffaele *et al.*, 2009), REM1.3 silencing was achieved by virus‐induced gene silencing (VIGS) with the tobacco rattle virus vector pTV00.

### Analysis of diversity and identification of candidate genes

We used a dataset of 213 624 SNPs on 1196 *A. thaliana* accessions (Atwell *et al.*, 2010; Horton *et al.*, 2012) to search for non‐synonymous mutations present in Lip‐0 and Rubezhnoe accessions but not in Col‐0, Nok‐1, Shahdara, and Rld‐2. The functional consequences of SNPs were predicted automatically using a script derived from the BioPython library (Cock *et al.*, 2009). For this, gene models from tair version 9 were used. Predictions were verified manually for the top candidates using expasy translate tool and http://signal.salk.edu/atg1001/ genome browser. LAZ5 closest homologues were identified using a BlastP search against phytozome 12.1 database. A protein sequence alignment of 350 positions was used as input for phylogenetic tree reconstruction by maximum likelihood method in phylogeny.fr (Dereeper *et al.*, 2008). Branch support was obtained by approximate likelihood‐ratio test using the WAG substitution model, four substitution rate categories, gamma = 1.794, proportion of invariants = 0.041, and gaps automatically removed from alignment. The input sequences, alignment, and resulting tree in newick format are provided in Data S2. Total RNA extraction was performed with Macherey‐Nagel Nucleospin RNA extraction kit following the manufacturer’s instructions. One µg of total RNA was used for cDNA synthesis in a 20‐µl reaction according to Roche Transcriptor Reverse Transcriptase protocol, using 0.5 µl of SuperScript II reverse transcriptase. Quantitative RT‐PCR reactions were performed on a LightCycler 480 apparatus (Roche Diagnostics, Basel, Switzerland, https://diagnostics.roche.com/global/en/home.html) as described in (Badet *et al.*, 2015), with primers given in Data S1. Relative gene expression corresponds to the ratio between the expression of *LAZ5* over expression of the reference gene *At2g28390*, with runs yielding Cp>32 for *At2g28390* excluded.

## AUTHORS CONTRIBUTIONS

AB, ON, and SR conceived the original screening and research plans; AB, ON, MM, and SR supervised the experiments; MB, LG, MK, and AL performed most of the experiments; AB and SR designed the experiments and analyzed the data; AB and SR conceived the project and wrote the article with contributions of all the authors; AB and SR agree to serve as the authors responsible for contact and ensure communication.

## CONFLICT OF INTEREST STATEMENT

The authors declare that they have no competing interests to disclose.

## Supporting information


**Figure S1.** Illustration of the Navautron box design and use. (a) Template for PMMA pieces making a Navautron box, with dimensions indicated in mm. (b) A Navautron bottom tray filled with 270 *A. thaliana* leaves inoculated by an agar plug colonized by *S. sclerotiorum*, at a start of an experiment. (c) Navautron phenotyping experiment running, with LED flashlights on.

**Data S1.** Representative PCR analysis for the genotyping of one T‐DNA mutant line. The file includes the list of the oligonucleotide primers used in this work and a map of the *LAZ5* locus showing the position of T‐DNA insertions (triangles) and primers (red arrows). Exons are shown are boxes, with the encoded protein domain labelled, introns are shown as plain lines. The expected size of PCR amplicons is indicated in red italic fonts. Picture of a representative agarose gel used to separate PCR products generated for screening three plants (#1–3) of line SALK_068316C. Sizes are indicated in base pairs. Primers used for each PCR reactions are indicated below the corresponding lane.

**Data S2.** Fasta sequence, multisequence alignment (FASTA format), and phylogenetic tree (NEWICK format) of LAZ5 and its 42 closest homologues (.txt file).

## Data Availability

Arabidopsis Genome Initiative locus identifiers for the genes in this article are as follows: At5g44870 (*LAZ5*); AT5G17880 (*CSA1*). The code and tutorials for the software presented in this manuscript are available from https://github.com/A02l01/INFEST
