# Supplementary material for: Rapid identification of an Arabidopsis NLR gene as a candidate conferring susceptibility to Sclerotinia sclerotiorum using time‐resolved automated phenotyping
Source: Plant J. 2020 Apr 21;103(2):903–17. doi: 10.1111/tpj.14747 (PMC7497225; doi:10.1111/tpj.14747)
Supplement: Supplementary file 1 — Figure S1. Illustration of the Navautron box design and use. (a) Template for PMMA pieces making a Navautron box, with dimensions indicated in mm. (b) A Navautron bottom tray filled with 270 A. thaliana leaves inoculated by an agar plug colonized by S. sclerotiorum, at a start of an experiment. (c) Navautron phenotyping experiment running, with LED flashlights on. [file TPJ-103-903-s001.pdf]

Adelin Barbacci*, Olivier Navaud, Malick Mbengue, Marielle Barascud, Laurence Godiard, Mehdi Khafif, Aline Lacaze, Sylvain Raffaele*

FIGURE S1

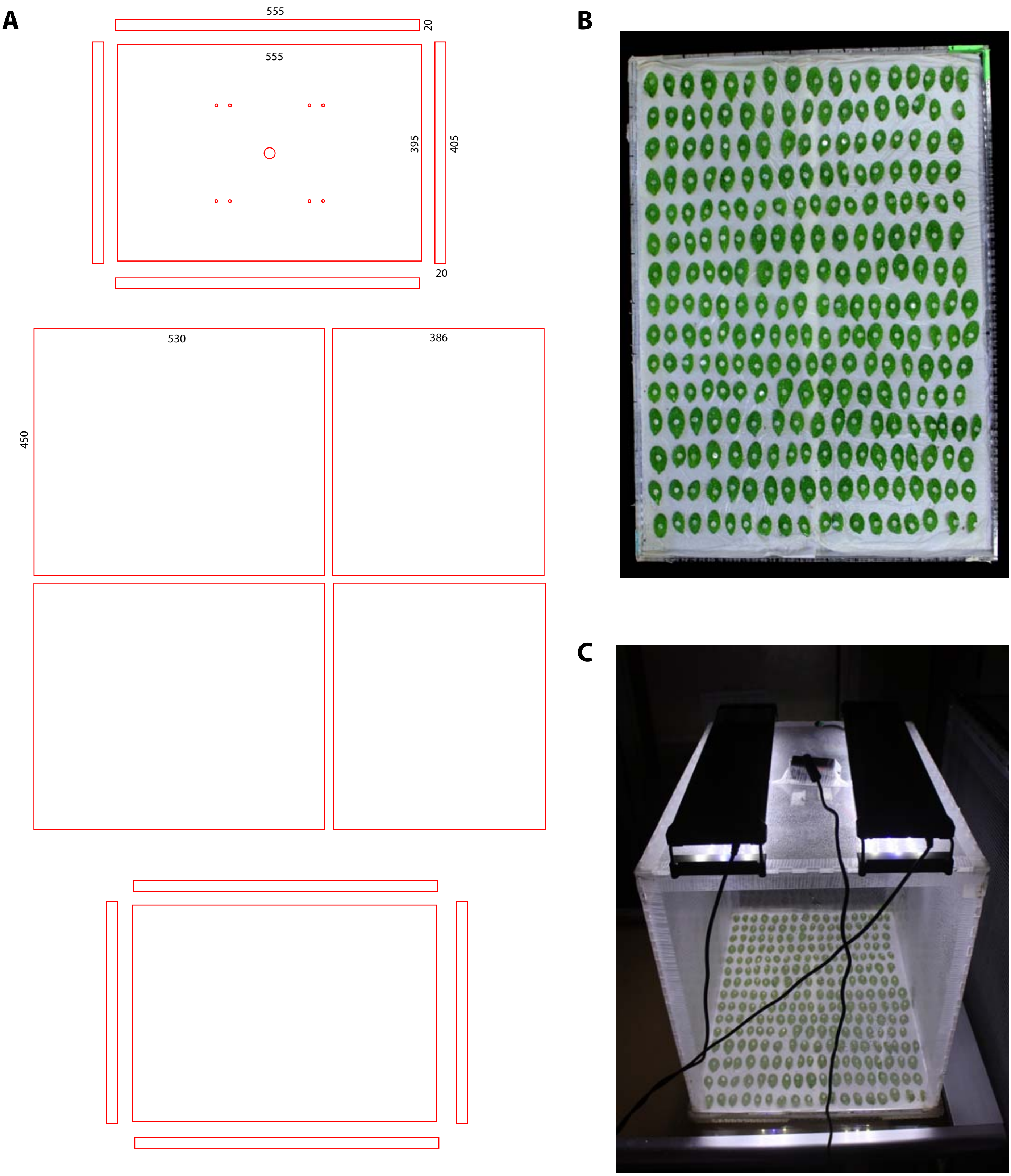

Figure S1. Illustration of the Navautron box design and use. (A) Template for PMMA pieces making a Navautron box, with dimensions indicated in mm. **(B)** A Navautron bottom tray filled with 270 *A. thaliana* leaves inoculated by an agar plug colonized by *S. sclerotiorum*, at the start of an experiment. **(C)** Nauvautron phenotyping experiment running, with LED flash lights on.
