# Supplementary material for: Rapid identification of an Arabidopsis NLR gene as a candidate conferring susceptibility to Sclerotinia sclerotiorum using time‐resolved automated phenotyping
Source: Plant J. 2020 Apr 21;103(2):903–17. doi: 10.1111/tpj.14747 (PMC7497225; doi:10.1111/tpj.14747)
Supplement: Supplementary file 2 — Data S1. Representative PCR analysis for the genotyping of one T‐DNA mutant line. The file includes the list of the oligonucleotide primers used in this work and a map of the LAZ5 locus showing the position of T‐DNA insertions (triangles) and primers (red arrows). Exons are shown are boxes, with the encoded protein domain labelled, introns are shown as plain lines. The expected size of PCR amplicons is indicated in red italic fonts. Picture of a representative agarose gel used to separate PCR products generated for screening three plants (#1–3) of line SALK_068316C. Sizes are indicated in base pairs. Primers used for each PCR reactions are indicated below the corresponding lane. [file TPJ-103-903-s002.pdf]

Rapid identification of an Arabidopsis NLR gene conferring susceptibility to Sclerotinia sclerotiorum using time-resolved automated phenotyping

Adelin Barbacci*, Olivier Navaud, Malick Mbengue, Marielle Barascud, Laurence Godiard, Mehdi Khafif, Aline Lacaze, Sylvain Raffaele*

Supplementary File 1.

The oligonucleotide primers used is this work were as follows:

Name	Sequence	Use
5.3LP	5'-GCGATTTCCGATTCCTCTATC-3'	Forward primer for genotyping of SALK_068316
5.3RP	5'-CCGCGGGTTAAATCCTAGTAG-3'	Reverse primer for genotyping of SALK_068316
LBb1.3	5'-ATTTTGCCGATTTCGGAAC-3'	T-DNA primer for genotyping SALK lines
5.1LP	5'-GAAGGTAAAAGCAAACGACCC-3'	Forward primer for genotyping of SALK_087262C
5.1RP	5'-GCTTGTGAAGCAAGTTCCTTG-3'	Reverse primer for genotyping of SALK_087262C, and *LAZ5* Q-RT-PCR
Laz5qFw	5'-CGTCGCATGTTTCTTCAAATC-3'	Forward primer for *LAZ5* Q-RT-PCR
csaLP	5'-CAACCTTCCAAGTAATGCTGC-3'	Forward primer for genotyping of SALK_023219C
csaRP	5'-GGCTGAAATTCCCGTTAAAAG-3'	Reverse primer for genotyping of SALK_023219C

Which hybridized as follows at the LAZ5 genomic locus:

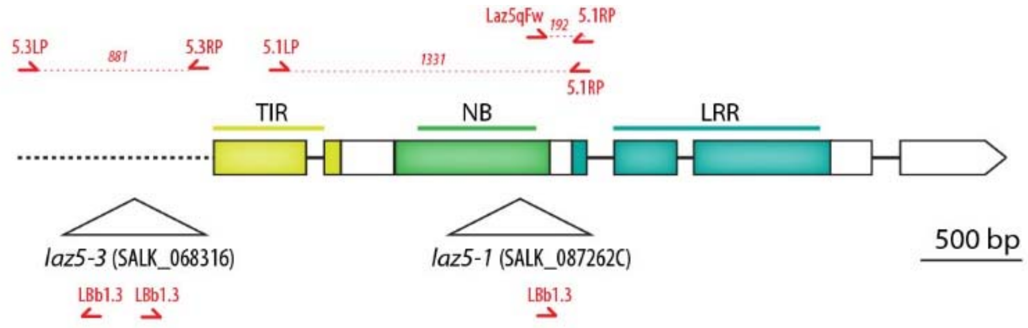

The PCR reaction used for mutant lines genotyping was 95°C 2 min; (95°C 30 sec.; 60°C 30 sec.; 72°C 1 min.) x 35 cycles; 72°C 5 min. Three primer pairs were tested on each plant as shown in the example below.

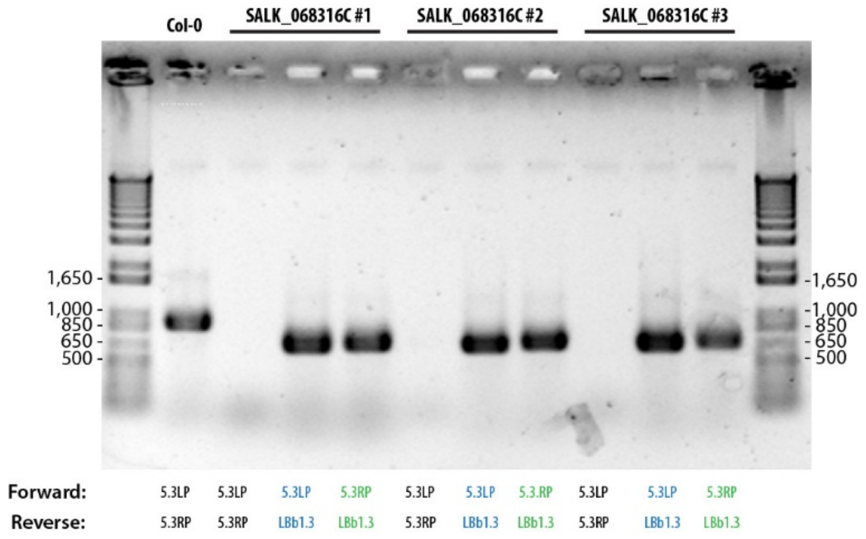

The PCR products obtained from one homozygous line were then sequenced using each of the three genotyping primers.
